# Is ethnicity a risk factor for mortality in major trauma? A single-centred cohort study

**DOI:** 10.1308/rcsann.2022.0097

**Published:** 2023-01-23

**Authors:** T Jennison, C Kulenkampff, J Lee, A Mahmood

**Affiliations:** University Hospitals Birmingham NHS Foundation Trust, UK

**Keywords:** Major trauma, Mortality, Ethnicity

## Abstract

**Introduction:**

Many studies have found varying health outcomes in patients from different minority ethnic groups. There has been limited research into the outcomes in major trauma dependent on ethnicity. The aim was to analyse whether ethnicity was an independent risk factor for 30-day mortality in patients presenting to a major trauma centre when adjusting for confounders.

**Methods:**

This was a retrospective review of all patients presenting to a single major trauma centre from 2010 to 2020. Data were collected on patient demographics and variables including mechanism and injury severity score. Logistic regression was used to determine significant predictors of mortality.

**Results:**

There were 10,668 data sets with ethnicity data; of these 9,098 were of White ethnicity, 1,143 were Asian and 427 were classified as Black. The 30-day mortality rate was 7.76% for White ethnicities, 6.91% for Asian ethnicity and 5.15% for people of Black ethnicity. On multivariate logistic regression, ethnicity (*p* = 0.076) was not associated with 30-day mortality. Age, Injury Severity Score (ISS), Probability of Survival (PS) score, shock and Glasgow Coma Scale (GCS; *p* < 0.001) were associated with 30-day mortality. White ethnicity had an odds ratio (OR) of mortality of 1.16 (95% confidence interval [CI] 0.658–2.040) (*p* = 0.609) compared with Black ethnicity and an OR of 0.74 (95% CI 0.546–1.001) (*p* = 0.050) compared with Asian patients. Black patients had an OR of mortality of 0.65 (95% CI 0.351–1.193) (*p* = 0.164) compared with the Asian population.

**Conclusion:**

Ethnicity is not a significant risk factor for 30-day mortality in trauma patients.

## Introduction

There has for some time been public concern that ethnicity plays a role in variation of health outcome following illness or injury, with worse outcomes experienced by minority ethnic groups. The COVID-19 health pandemic has recently brought this to light, with case studies demonstrating worse outcomes in Black and Asian ethnic groups.^1^ In addition, the MBRRACE-UK report found that women from minority ethnic backgrounds were more likely to die in childbirth than White women.^2^ Health inequalities in people with a minority ethnic background is a complex issue in a heterogeneous group, with multiple variables accounting for varying outcomes.^3,4^

Although there is no consensus in the literature on the effect of ethnicity on mortality, several US studies have demonstrated higher mortality in Black populations.^5,6^ There is also a paucity of data on the effect of social deprivation on the outcomes of major trauma. Research into the effect of ethnicity on the outcomes of major trauma is limited, and the majority of these publications are from the USA.^4,7^

With the limited data available on outcomes in major trauma, and the concerning findings seen in response to COVID-19 and childbirth, it is vital that health outcomes from major trauma are assessed for the impact of ethnicity.

The aim of this cohort study was to determine mortality rates for different ethnic groups presenting to a single UK major trauma centre over a 10-year period and to examine any differences in mortality rates when adjusting for confounders.

## Methods

A retrospective evaluation of all patients presenting to a single major trauma centre from 2010 to 2020 was undertaken. Data were collected prospectively by the major trauma team, and from medical records on discharge. This data collection forms part of the Trauma Audit and Research Network (TARN) data collection and therefore ethical approval was not required.^8^ All patients that were TARN eligible were included. The TARN criteria are trauma patients irrespective of age, who fulfil one of the following criteria: (a) in hospital for more than three overnight stays; (b) admitted to a critical care area (regardless of length of stay); (c) transferred out for specialist care or repatriation; (d) transferred in for specialist care or repatriation; (e) deaths and whose isolated injuries must meet a specified criterion. Patients were included if they had a recorded ethnicity on the data collection.

Data were collected on age, gender, mechanism of injury and Glasgow Coma Scale (GCS), and the presence of shock on arrival, Injury Severity Score (ISS), Probability of Survival (PS) and postcode.

The ISS is an anatomical score that measures the overall severity of injured patients. All injuries are scored between 1 (minor injury) and 6 (an injury that is incompatible with life). The ISS is calculated by adding the squares of the three worst injured body regions.^9^

The Index of Multiple Deprivation (IMD) score is a unique measure of relative deprivation. It is produced based on seven different domains of deprivation: income deprivation, employment deprivation, education skills and training deprivation, health deprivation and disability, crime, barriers to housing and services, and living environment deprivation. It ranks every small area in England from 1 (most deprived area) to 32,844 (least deprived area). These are then divided into deciles with those in decile one being the most deprived and those in decile ten least deprived. Patients postcodes were linked to the IMD to give every patient a score from 1 to 10.^10^

Ethnicity was divided into Asian, Black or White. Those of mixed race or where the race was uncertain were excluded. These three main groups were chosen because further subdivision would have caused several groups to have small numbers making meaningful statistical analysis impossible.

The PS model is calculated for each patient and gives a percentage calculation for probability of survival. This takes into account the ISS, GCS, Charlson comorbidity score, age and gender.^8^

### Statistical analysis

Statistical analysis was undertaken using Stata (version 15). Participant demographics and variables on presentation were summarised using means and standard deviations for continuous variables, and numbers and percentages for categorical variables. Demographic characteristics were compared between the three ethnic groups using analysis of variance for continuous variables and the chi-squared test for categorical variables.

Logistic regression was performed initially undertaking univariate and then multivariate analysis. Results are expressed with odds ratios (OR), 95% confidence intervals (CI) and *p*-values.

## Results

There were 10,668 data sets with ethnicity data; of these 9,098 were White, 1,143 were Asian and 427 were classified as Black. The 30-day mortality rate was 7.56% (807). The mortality rate was 7.76% for White ethnicities, 6.91% for Asian, and 5.15% for Black ethnicities.

For the 9,098 White patients, the median age was 61.5 (interquartile range [IQR] 41.2–80.4) and 62.1% were female. The mean GCS was 13.63 and the mean ISS was 16.34 with a PS of 90.86. For the 1,143 Asian patients, the median age was 42.4 (IQR 26.3–65.4) and 25.6% were female. The mean GCS was 13.3 and the mean ISS was 17.49 with a PS of 92.04. For the 427 Black patients, the median age was 34.0 (IQR 24.3–54.0) and 17.8% were female. The mean GCS was 13.4 and the mean ISS was 17.19 with a PS of 92.34.

The mean IMD score was 2.03 in Black patients, 2.70 in Asian patients and 4.09 in White patients (*p* < 0.0001), indicating that those of Black and Asian ethnicity were significantly more likely to be more deprived (Table 1).

**Table 1:** Demographics based on ethnic group

Risk factor	Asian	Black	White	*p*-value
*N*	1,143	427	9,098	
Age	42.4	34.0	61.5	0.0001
ISS	17.49 (10.85)	17.19 (11.14)	16.34 (10.64)	0.0011
Social deprivation	2.70 (2.21)	2.03 (1.69)	4.09 (2.73)	0.0001
Female, *n* (%)	292 (25.55)	76 (17.80)	3,485 (62.09)	0.0001
Shock	21.26%	23.19%	20.53	0.3828
GCS	13.31	13.40	13.63	0.0337
PS	92.04	92.34	90.86	0.0277

GCS = Glasgow Coma Scale; ISS = Injury Severity Score; PS = Probability of Survival. The mean age is reported in the table.

Overall White patients were found to be significantly older, had a lower ISS, a higher social deprivation score, were more commonly female, had a lower PS score, and were more likely to have a blunt mechanism of injury such as a low-energy fall (Table 2).

**Table 2:** Mechanism of injury

Mechanism	Asian		Black		White		*p*-value
	*n*	%	*n*	%	*n*	%	
*N*	1,143		427		9,098		
Blast	1	0.09	3	0.70	17	0.19	0.043
Blow with weapon	2	0.17	2	0.47	4	0.04	0.003
Blow without weapon	128	11.19	38	8.90	512	5.63	0.001
Burn	0	0	0	0	5	0.05	0.353
Crush	7	0.61	4	0.94	79	0.87	0.657
Fall <2m	381	33.33	106	24.82	4,589	50.44	0.001
Fall >2m	130	11.37	35	8.20	1,145	12.59	0.016
Other	14	1.22	3	0.70	88	0.97	0.591
Shooting	12	1.05	29	6.79	25	0.27	0.001
Stabbing	114	9.97	82	19.20	312	3.43	0.001
Vehicle incident/collision	354	30.97	125	29.27	2,322	25.52	0.001

In the Asian population, the most common mechanism of injury was a fall of less than 2m (33.33%) followed by vehicle collision (30.97%) and a fall of greater than 2m (11.37%). In the Black population, the most common mechanism of injury was motor vehicle collision (29.27%), followed by a fall less than 2m (24.82%) and stabbing (19.20%). In the White population, the most common presentation was due to a fall of less than 2m (50.44%), vehicle collision (25.52%) and a fall of greater than 2m (12.59%). Analysing the proportion of different mechanisms, shootings, stabbings, blasts and blows with weapons were all significantly more common in the Black population. Blows without weapons were significantly more common in the Asian and Black populations compared with the White population. Falls of less than 2m with significantly more common in the White population, and falls of greater than 2m more common in the White and Asian populations.

On univariate logistic regression ethnicity was not associated with mortality (OR 1.09, 95% CI 0.97–1.23). Social deprivation was associated with mortality (OR 1.05, 95% CI 1.02–1.07). Risk factors found to be significantly associated with mortality were age (OR 1.03, 95% CI 1.02–1.03), GCS (OR 0.79, 95% CI 0.77–0.80), injury mechanism (OR 0.96, 95% CI 0.93–0.99), ISS (OR 1.06, 95% CI 1.05–1.07), shock (OR 1.87, 95% CI 1.60–2.19) and PS (OR 0.95, 95% CI 0.94–0.95).

On multivariate logistic regression, ethnicity (*p* = 0.076), social deprivation (*p* = 0.953), injury mechanism (*p* = 0.079) and gender (*p* = 0.055) were not associated with mortality. Age (OR 1.04, 95% CI 1.04–1.05), ISS (OR 1.03, 95% CI 1.02–1.04), PS score (OR 0.98, 95% CI 0.97–0.99), shock (OR 1.90, 95% CI 1.56–2.31) and GCS (OR 0.84, 95% CI 0.80–0.88) were associated with 30-day mortality (all *p*-values < 0.001) (Table 3).

**Table 3:** Logistic regression of risk factors for mortality

Risk Factor	Univariate			Multivariate S		
	Odds ratio	95% CI	*p*-value	Odds ratio	95% CI	*p*-value
Age	1.03	1.02–1.03	0.000	1.045	1.04–1.05	0.000
Gender	1.01	0.87–1.17	0.911	1.21	1.00–1.46	0.055
GCS	0.79	0.77–0.80	0.000	0.84	0.80–0.88	0.000
Mechanism	0.96	0.93–0.99	0.004	0.96	0.92–1.00	0.079
ISS	1.06	1.05–1.07	0.000	1.03	1.02–1.04	0.000
Shock	1.87	1.60–2.19	0.000	1.90	1.56–2.31	0.000
Ethnicity	1.09	0.97–1.23	0.147	0.87	0.75–1.01	0.076
Index for multiple deprivation	1.05	1.02–1.07	0.001	1.00	0.97–1.03	0.953
Probability of Survival	0.94	0.94–0.95	0.000	0.98	0.97–0.99	0.000

GCS = Glasgow Coma Scale; ISS = Injury Severity Score

When performing logistic regression modelling specifically looking at the differences in mortality between ethnic groups when adjusting for confounders White ethnicity had an OR of mortality of 1.159 (95% CI 0.658–2.040) (*p* = 0.609) compared with Black ethnicity. Black patients had an OR of mortality of 0.647 (95% CI 0.351–1.193) (*p* = 0.164) compared with the Asian population, and White people had an OR of mortality of 0.739 (95% CI 0.546–1.001) (*p* = 0.050) compared with Asian patients.

## Discussion

This study demonstrated that ethnicity was not a significant factor for mortality following major trauma. The significant risk factors for mortality were age, ISS, PS score, shock and GCS. This study is the first to assess mortality in major trauma based on ethnicity.

Major trauma can affect patients from young to old. Trauma results in approximately 16,000 deaths per year in England and Wales, and is the leading cause of death among children and adults aged less than 44 years.^8^

Recent studies have demonstrated increased mortality rates in minority ethnic groups during childbirth and from COVID-19.^1,2^ There are many socio-economic differences in England based on ethnicity.^3^ When analysing the demographics of patients with major trauma, this study found that overall White patients were older and more commonly female, had a higher social deprivation score and a lower PS score. These factors were taken into consideration when mortality was analysed. The mechanism of injury was more commonly a fall from less than 2m in White patients. Although the proportion of stabbings was higher in the Black population (six times higher by proportion), the overall number of stabbings was highest in the White population.

Although this study did not demonstrate that ethnicity was a risk factor for mortality, others have found contrasting results. The majority of these publications were from a US setting. A meta-analysis by Hicks *et al* found that Black race was associated with an increased odds of death when compared with White ethnic groups, but found no differences compared with Hispanics. In this cohort, 61.7% of patients presented with a ISS of ≥9; however, ISS was not stratified according to ethnicity.^7^ Conversely, Arthur *et al* factored ISS into their analysis, finding that the increased risk for mortality in Black trauma patients was especially apparent in those with mild to moderate injuries, whereas the increase mortality seen in the Asian subgroup was most recognised in severely injured patients.^6^ Duong *et al* in a study of over one million patients in the trauma quality improvement programme found that Black and Asian trauma patients had a higher associated risk of death, after adjusting for ISS, when compared with White patients, in all regions except the northeast USA.^11^ Glance *et al*, after accounting for ISS, and Haider *et al* (in their meta-analysis) reported that racial disparities were due to the fact that Black patients were more likely to be treated in lower quality hospitals than White patients rather than as a result of other factors.^4,5^ This is a situation that does not occur in England due the development of major trauma networks, and the continual audit and monitoring of outcomes at major trauma centres and units.

In a further study in the USA, outcomes in vascular trauma have been shown to be due to the mechanism of injury rather than ethnicity in young patients, but in elderly populations race has been associated with worse outcomes. The reasons for this are unclear, but may reflect deprivation.^12^

This study did not demonstrate socio-economic status to be associated with mortality in major trauma. By contrast to this, some studies from the USA have demonstrated increased mortality from major trauma with lower incomes.^7^ Again, this may be explained by differences in healthcare provision between the two countries.

### Study limitations

There are several limitations to this study including that it is a single centre. This study only breaks ethnicity down into Black, Asian and White. Ethnicity is more complex than this, and differences in social demographics and health outcomes have been reported between different ethnic groups.^2-4^ Because of the size of the study population, it was decided to use these broad groups to be able to produce meaningful results. The study also does not consider overall population demographics and therefore cannot make conclusions on the incidence of these injuries in the population. It also does not consider out-of-hospital deaths, which may have affected the overall findings. A further limitation is the disparity in the numbers in the groups. This reflects the different numbers of people of different ethnicities in the general population.

Despite these limitations, this is the largest study on the effect of social deprivation and ethnicity in mortality in major trauma in the UK. It also takes into account potential confounders including using the PS score to compare survivorship in different ethnic groups.

## Conclusion

Ethnicity is not a significant risk factor for 30-day mortality in trauma patients. Any differences in mortality rates can be accounted for by differences in in patient demographics including comorbidities and the severity of injuries on presentation.

A larger study population and geographic differences across the UK may help to confirm our findings and may help to tease out differences within ethnic subsets/populations.
